# IgA Vasculitis with Nephritis in Adults: Histological and Clinical Assessment

**DOI:** 10.3390/jcm10214851

**Published:** 2021-10-22

**Authors:** Lingyun Lai, Shaojun Liu, Maria Azrad, Stacy Hall, Chuanming Hao, Jan Novak, Bruce A. Julian, Lea Novak

**Affiliations:** 1 Division of Nephrology, Fudan University Huashan Hospital, Shanghai 200040, China; lailingyun@fudan.edu.cn (L.L.); liushaojun@fudan.edu.cn (S.L.); chuanminghao@fudan.edu.cn (C.H.); 2 Department of Microbiology, University of Alabama at Birmingham, Birmingham, AL 35294, USA; shall@uab.edu (S.H.); jannovak@uab.edu (J.N.); 3 Department of Nutrition, University of Alabama at Birmingham, Birmingham, AL 35294, USA; mazrad@ches.ua.edu; 4 Department of Medicine, University of Alabama at Birmingham, Birmingham, AL 35294, USA; bjulian@uabmc.edu; 5 Department of Pathology, University of Alabama at Birmingham, Birmingham, AL 35294, USA

**Keywords:** IgA vasculitis, nephritis, kidney biopsy

## Abstract

Patients with IgA vasculitis (IgAV), an immune complex-mediated disease, may exhibit kidney involvement—IgAV with nephritis (IgAVN). The kidney-biopsy histopathologic features of IgAVN are similar to those of IgA nephropathy, but little is known about histopathologic disease severity based on the interval between purpura onset and diagnostic kidney biopsy. We assessed kidney histopathology and clinical and laboratory data in a cohort of adult patients with IgAVN (n = 110). The cases were grouped based on the interval between the onset of purpura and kidney biopsy: Group 1 (G1, <1 month, n = 14), Group 2 (G2, 1–6 months, n = 58), and Group 3 (G3, >6 months, n = 38). Glomerular leukocytes were more common in G1 than in the other groups (*p* = 0.0008). The proportion of neutrophils among peripheral-blood leukocytes was the highest in the patients biopsied within a month after onset of purpura (G1: 71 ± 8%). In the patients with an interval >6 months, the neutrophil proportion was lower, 60%. Moreover, the glomerular mesangial proliferation score correlated with the serum total IgA concentration (*p* = 0.0056). In conclusion, IgAVN patients biopsied <1 month from purpura onset showed an elevated percentage of blood neutrophils and glomerular leukocytes, consistent with an acute-onset inflammatory reaction. In all IgAVN patients, the mesangial proliferation score correlated with the serum IgA level.

## 1. Introduction

IgA vasculitis (IgAV), formerly known as Henoch–Schönlein purpura, is a systemic immune complex-mediated, small-vessel leukocytoclastic vasculitis. It is characterized by nonthrombocytopenic palpable purpura and/or arthritis, and abdominal pain [1]. IgAV, the most common vasculitis in children, is often a self-limiting and benign disease that spontaneously resolves.

A minority of pediatric IgAV patients exhibits kidney involvement—IgAV with nephritis (IgAVN)—usually 4–6 weeks after the onset of purpura [2,3,4,5]. The kidney-biopsy histopathologic features of IgAVN are similar to those of IgA nephropathy (IgAN), including glomerular IgA1-containing immunodeposits [1,2,4,6,7,8,9,10,11].

IgAV in adults has a more severe course and poor outcome due to the high frequency of glomerulonephritis, i.e., IgAVN—the most serious complication of this vasculitis. However, there is limited information about the histopathologic disease severity as related to disease onset.

In this study, we assessed the kidney histopathology and clinical and laboratory data in a large cohort of adult patients with IgAVN whose diagnostic kidney biopsies had been performed at different intervals after the onset of purpura.

## 2. Materials and Methods

### 2.1. IgAVN Patients

This is a retrospective study of 110 adult patients with IgAVN. All patients underwent a diagnostic kidney biopsy between 2002 and 2013 at the Department of Nephrology, Huashan Hospital, Fudan University, Shanghai, China. The diagnosis of IgAVN was based on the documented hematuria and proteinuria associated with a characteristic purpuric eruption, or abdominal or joint pain. IgA as the predominant mesangial immunoglobulin as per immunofluorescence microscopy in kidney biopsy specimens was required for inclusion in the study. We excluded patients whose biopsies contained <8 glomeruli, a number considered inadequate for appropriate histopathologic scoring (12), as detailed below.

The appearance of purpura defined the onset of IgAV. The 110 patients were divided into three groups based on the interval between purpura onset and diagnostic kidney biopsy: Group 1, <1 month (n = 14); Group 2, 1–6 months (n = 58); and Group 3, >6 months (n = 38; mostly >1 year, with the longest interval 13 years). All biopsies were performed for subjects who exhibited proteinuria in an outpatient setting (>0.5 g/24 h or urinary albumin/creatinine ratio >300 mg/g). The clinical features, laboratory data from the time of biopsy, and histopathologic findings in the biopsy specimens were retrospectively compiled for each group. However, no data from the outpatient follow-up visits since the onset of purpura or after kidney biopsy were available for this study. No personal identification information was collected. The study was approved by the ethics board of Huashan Hospital, Fudan University, Shanghai, China.

### 2.2. Clinical and Demographic Data

All clinical, laboratory, and demographic data were collected at the time of the kidney biopsy. The demographic data included age, gender, and comorbidities such as hypertension, diabetes mellitus, and cardiovascular disease. The characteristics of IgAVN included skin rash, gastrointestinal and joint manifestations, and kidney involvement. Proteinuria was evaluated by a 24-h urine measurement, hematuria was defined as 22 or more red blood cells (RBC) per microliter of urine (microscopic), or visible hematuria (macroscopic). The serum albumin, creatinine, urea nitrogen, uric acid, complement C3, and total IgA levels were measured at the time of kidney biopsy in the central clinical laboratory of Huashan Hospital. The number of urinary RBC had been determined by using a Sysmex UF-1000i analyzer (Siemens, Germany). Peripheral blood cell profiling was performed using a Sysmex xn-2000 analyzer (Siemens, Germany) and expressed as the total number of leukocytes and relative proportions of neutrophils and eosinophils (as % of total leukocytes).

### 2.3. Kidney Pathology

**Figure 1:**
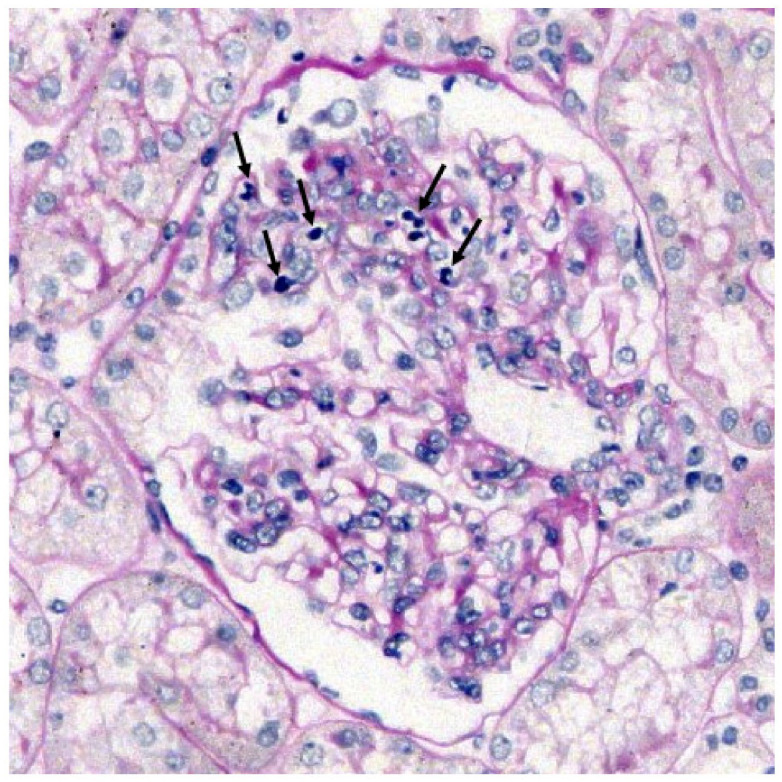
Example of a glomerulus of a patient with IgAVN exhibiting mesangial proliferation and glomerular leukocytes. Representative leukocytes are marked by arrows. PAS stain, magnification 400×.

Two pathologists examined and graded the histopathological changes. To evaluate the glomerular mesangial-cell proliferation, the cellularity of each glomerulus was graded as per the Oxford classification [12] (<4 mesangial cells/mesangial area = 0; 4–5 mesangial cells/mesangial area = 1; 6–7 mesangial cells/mesangial area = 2; >8 mesangial cells/mesangial area = 3), and a mean mesangial score was calculated for each biopsy. Mesangial Score: sum of grades divided by number of glomeruli (excluding globally sclerotic glomeruli). Crescents (%): number of glomeruli with crescents divided by number of glomeruli × 100. Leukocyte infiltration of glomeruli was considered significant when five or more polymorphonuclear and mononuclear cells per glomerulus were observed using the periodic-acid Schiff-stained tissue sections [11,12] (Figure 1).

### 2.4. Statistical Analyses

Normally distributed variables are expressed as the mean ± standard deviation (SD) and were compared using one-way analysis of variance (ANOVA) or Student’s *t*-test. Nonnormally distributed variables are expressed as the median with interquartile range and were compared using the rank sum test. Categorical variables are expressed as percentages and compared using Pearson’s chi-square test or Fisher’s exact test. All tests were two-tailed, and statistical significance was defined as *p* < 0.05. The SPSS statistical software program (version 15.0, SPSS Inc., Chicago, IL, USA) was used for all analyses.

## 3. Results

### 3.1. Baseline Clinical Data at the Time of Kidney Biopsy

IgAVN patients (n = 110) in this study had a mean age of 36.5 ± 16.0 years at the time of kidney biopsy and consisted of 50 males and 60 females (Table 1). All patients presented with cutaneous purpura on at least one occasion; purpura was associated with arthralgia in 30 cases (26%) and with arthralgia and abdominal pain in 31 cases (27%). At the time of kidney biopsy, proteinuria ≥0.30 g/24 h was detected in 105 patients (92%) and 73 patients had proteinuria ≥1 g/24 h (64%). Five patients with proteinuria ≥0.5 g/24 h originally measured in the outpatient clinic had proteinuria <0.30 g/24 h later on admission to the hospital for kidney biopsy, likely due to prior treatment with an angiotensin-converting enzyme inhibitor (ACEi) and/or angiotensin receptor blocker (ARB).

Microscopic hematuria was observed in 86 patients (78%). Hypertension was present in 23 patients (21%) and 4 patients (4%) had diabetes mellitus (DM). Seven patients (6%) had reduced kidney clearance function, with an eGFR <60 mL/min/1.73 m^2^ as per the MDRD formula. Twenty-seven patients (25%) had received an ACEi and/or an ARB before kidney biopsy.

### 3.2. Kidney Histopathology and Kidney Function

The light-microscopic features for this cohort are summarized in Table 2. The patients had a mean mesangial score of 1.1 (range 0.3–2.4); 18% exhibited segmental sclerosis; 3%, global sclerosis; 25%, glomerular adhesion; 20%, glomerular leukocytes; 43%, tubular atrophy; 40%, interstitial fibrosis; 39%, interstitial leukocytes; and 9%, crescents. Kidney function was worse in the subjects with crescents than in those without crescents (serum creatinine: 0.91 ± 0.43 mg/dL vs. 0.76 ± 0.20 mg/dL, *p* = 0.038; eGFR: 93 ± 32 mL/min/1.73 m^2^ vs. 109 ± 28 mL/min/1.73 m^2^, *p* = 0.009).

### 3.3. Histopathology of Kidney Biopsy Specimens with Different Intervals between Purpura Onset and Diagnostic Kidney Biopsy

We next assessed whether the kidney pathology findings differed based on the interval between purpura onset and diagnostic kidney biopsy. The MEST-C scores (12) were calculated (Table 3). Using ANOVA and Student’s *t*-test, the only significant difference between the groups was for M1 between Group 2 and Group 3 (*p* = 0.006). Furthermore, glomerular leukocytes were more common in Group 1 (57%) compared to Group 2 and Group 3 (*p* = 0.0008) (Table 3). Thus, IgAVN patients with kidney biopsy less than one month after purpura onset more frequently had leukocytes in the glomeruli. Furthermore, M1 was more common in Group 3 than in Group 2.

### 3.4. Neutrophils in Peripheral Blood in Patients with Different Intervals between Purpura Onset and Diagnostic Kidney Biopsy

Patients in Groups 1, 2, and 3 had a similar age, gender representation, mean 24-h proteinuria, hematuria, mean eGFR, and frequency of ACEi/ARB treatment. However, the percentage of neutrophils in the circulating leukocytes differed between groups (Group 1: 71 ± 8%; Group 2: 68 ± 11%; and Group 3: 60 ± 12%; *p* = 0.001), being the highest in patients biopsied within a month after onset of purpura (Table 3). When we evaluated the neutrophils to lymphocytes ratio (NLR), there was no significant difference for any comparison of the groups (Table 3).

### 3.5. Association of Serum Total IgA Concentration and Mesangial Proliferation

**Figure 2:**
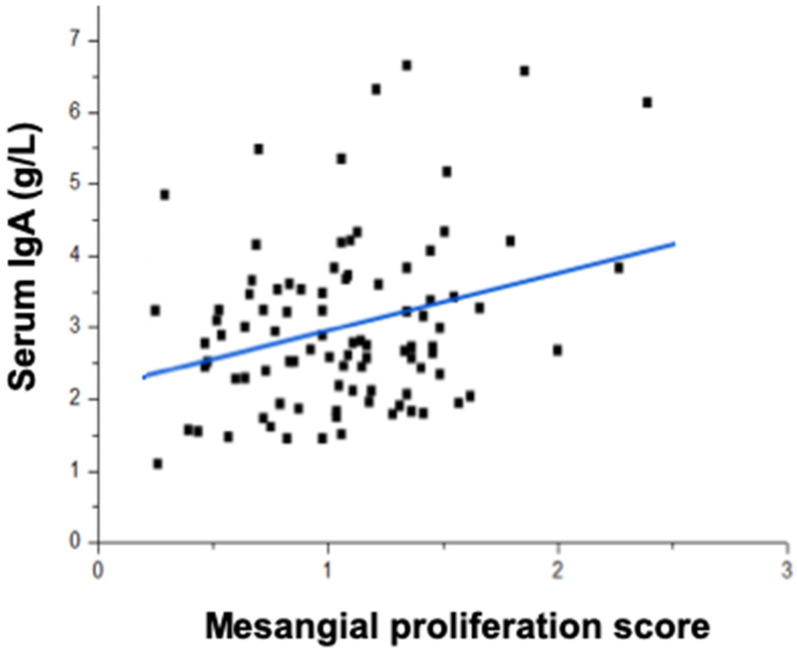
Glomerular mesangial proliferation correlates with serum total IgA level. Serum total IgA levels correlate with the mesangial proliferation score in patients with IgAVN. The line shows a linear fit (*p* = 0.0056).

The serum total IgA concentration positively correlated with the glomerular mesangial-proliferation score (*p* = 0.0056) (Figure 2).

## 4. Discussion

IgAV is the most common vasculitis in children but it can also occur in adults. IgAV spontaneously resolves in about 94% of children and 89% of adults [13], although some IgAV patients exhibit kidney involvement (IgAVN). The disease mechanisms of IgAVN and IgAN are thought to be closely related [6,9]. Both diseases exhibit glomerular IgA immunodeposits with co-deposits of complement C3 [13,14,15,16,17,18]. The IgA immunodeposits are of the IgA1 subclass [18,19,20].

Pathogenesis of both IgAN and IgAVN is thought to occur through a multi-hit process [5]. This process includes the production of galactose-deficient IgA1 (Gd-IgA1) [21,22,23,24,25,26,27,28,29], generation of circulating IgG autoantibodies specific for Gd-IgA1 [29,30,31,32,33,34], formation of pathogenic Gd-IgA1-containing immune complexes [30,35,36,37,38,39,40,41,42,43], and the subsequent mesangial deposition of these immune complexes resulting in glomerular injury [34,44]. These conclusions are supported by multiple lines of evidence. For example, serum levels of Gd-IgA1 and the corresponding IgG autoantibodies are associated with a faster decline in kidney function in patients with IgAN [45,46,47]. Moreover, IgA-containing glomerular immunodeposits are enriched for Gd-IgA1 glycoforms [48,49] and the corresponding IgG autoantibodies [33]. In both IgAN and IgAVN, Gd-IgA1 is produced by IgA1-secreting cells due to dysregulation of key glycosyltransferases [29,50,51].

Circulating levels of Gd-IgA1 and Gd-IgA1-specific IgG autoantibodies are elevated in patients with IgAVN but not in patients with IgAV [29], supporting the hypothesis that IgAVN and IgAN share pathogenetic components. IgAVN patients have the onset of disease defined by purpura, with kidney involvement developing with 4–6 weeks later [5,13,17]. However, there is a limited information about histopathologic disease severity in relation to disease onset.

In this study of 110 adult patients with IgAVN, we assessed histopathologic disease severity based on interval between purpura onset and diagnostic kidney biopsy and correlated the findings with clinical and laboratory data. IgAVN patients biopsied <1 month since the onset of purpura more commonly had glomerular leukocytes and had the highest percentage of neutrophils among peripheral-blood leukocytes. These findings are consistent with an acute-onset inflammatory reaction in patients with IgAVN who were biopsied <1 month after the onset of purpura. Moreover, serum total IgA concentration correlated with the glomerular mesangial proliferation score.

A limitation of this study is the relatively small number of patients from a single center who were of single ethnicity. The patients were followed regularly for up to 6 months after the appearance of purpura at their respective local-area hospitals. However, these data were not been available for this study. This limitation implies that these findings need to be assessed in other cohorts and different ethnic groups, ideally with regular follow-up after the onset of purpura until biopsy as well as after biopsy.

Our study demonstrates a chronological association between leukocytic infiltration and glomeruloproliferative responses in IgAVN patients. These findings raise a question whether patients with IgAV should be followed frequently after the onset of purpura to detect nephritis in the early stages and whether assessment of biomarkers, such as Gd-IgA1- and Gd-IgA1-specific IgG autoantibodies, should be included.

## Figures and Tables

**Table 1:** Clinical characteristics of 110 patients with IgAVN.

Characteristics	Values
Age (years, mean ± SD)	36.5 ± 16.0
Males (%)	50 (45)
Subjects with hypertension (%)	23 (21)
Subjects with diabetes mellitus (%)	4 (4)
Serum creatinine (mg/dL) mean (95% CI)	0.85 (0.78, 0.92)
eGFR (mL/min/1.73 m^2^, mean ± SD)	110 ± 43
Subjects on ACEi/ARB before kidney biopsy (%)	27 (25)

Abbreviations: SD, standard deviation; CI, confidence interval; eGFR, estimated glomerular filtration rate; ACEi, angiotensin-converting enzyme inhibitor; ARB, angiotensin receptor blocker.

**Table 2:** Summary of the kidney pathology findings for 110 patients with IgA vasculitis with nephritis.

Characteristics	Values
Mesangial score: mean (95% CI)	1.1 (1.02–1.17)
Segmental sclerosis (%)	18
Global sclerosis (%)	4
Glomerular adhesion (%)	26
Glomerular leukocytes (%)	20
Tubular atrophy (%)	43
Interstitial fibrosis (%)	40
Interstitial leukocytes (%)	39
Crescents (%)	9

Abbreviations: CI, confidence interval.

**Table 3:** Characteristics of 110 patients with IgAVN grouped based on the interval between purpura onset and diagnostic kidney biopsy.

Characteristics	Group 1 ^a^	Group 2	Group 3	*p* Value
	(n = 14)	(n = 58)	(n = 38)	
Age (yr) ^c^, (mean ± SD)	40.5 ± 19.3	36.8 ± 16.0	34.5 ± 14.9	0.47 ^b^
Male gender (% male)	6 (43)	28 (48)	16 (42)	0.82
Hypertension (%)	4 (29)	12 (21)	7 (18)	0.73
Diabetes mellitus (%)	2 (14)	1 (2)	1 (3)	0.07
ACEi/ARB before biopsy (%)	10 (19)	6 (32)	11 (28)	0.74
Proteinuria (g/24 h) (range)	2.23 (1.11, 4.68)	1.39 (0.87, 2.29)	1.13 (0.59, 1.93)	0.08
Urinary RBC (number/μL; mean ± SD)	667 ± 885	369 ± 602	405 ± 660	0.29
SCr (mg/dL; mean ± SD)	0.97 ± 0.34	0.83 ± 0.40	0.83 ± 0.29	0.42
eGFR (mL/min/1.73 m^2^; mean ± SD)	91 ± 29	113 ± 49	112 ± 36	0.28
WBC (×10^9^/L; mean ± SD)	11.1 ± 7.0	9.3 ± 3.6	8.5 ± 3.4	0.13
Neutrophils (% of WBC; mean ± SD)	71 ± 8	68 ± 11	60 ± 12	1 vs. 2 = 0.3067 1 vs. 3 = 0.0039 2 vs. 3 = 0.0008
Eosinophils (% of total WBC; mean ± SD)	2 ± 4	1 ± 2	2 ± 3	0.40
Serum albumin (g/dL; mean ± SD)	3.3 ± 0.6	3.5 ± 0.6	3.7 ± 0.5	0.14
Serum total IgA (g/L; mean ± SD)	3.1 ± 1.2	3.0 ± 1.3	2.9 ± 1.0	0.86
Serum C3 (g/L; mean ± SD)	1.17 ± 0.30	1.09 ± 0.24	1.10 ± 0.25	0.57
Glomeruli with leukocytes (%)	8 (57)	6 (10)	8 (21)	0.0008 ^b^
NLR (mean ± SD) c	2.7 ± 0.8	2.6 ± 1.5	2.1 ± 1.4	0.09 ^b^
MEST-C score				
M1 (%)	71	68	95	0.0075 ^b^ 2 vs. 3 = 0.006
E1 (%)	14	42	30	0.106 ^b^
S1 (%)	21	14	24	0.397 ^b^
T1/T2 (%)	7	5	14	0.344 ^b^
C1/C2 (%)	57	49	60	0.598 ^b^

^a^ 110 cases were divided into three groups: Group 1, <1 month; Group 2, 1–6 months; and Group 3, >6 months. ^b^ Fisher’s Exact for proportions. ^c^ Abbreviations: yr, years; SD, standard deviation; SCr, serum creatinine; CI, confidence interval; eGFR, estimated glomerular filtration rate; ACEi, angiotensin-converting enzyme inhibitor; ARB, angiotensin receptor blocker; NLR, neutrophils to lymphocytes ratio; RBC, red blood cells; WBC, white blood cells (leukocytes) in peripheral blood. MEST-C scores were determined as described previously (12). M: mesangial hypercellularity; E: endocapillary hypercellularity; S: segmental glomerulosclerosis; T: tubular atrophy/interstitial fibrosis; C: cellular/fibrocellular crescents.

## Data Availability

Not Applicable.
